# Diet Quality and Diet Diversity in Eight Latin American Countries: Results from the Latin American Study of Nutrition and Health (ELANS)

**DOI:** 10.3390/nu11071605

**Published:** 2019-07-15

**Authors:** Georgina Gómez, Regina Mara Fisberg, Ágatha Nogueira Previdelli, Cristiane Hermes Sales, Irina Kovalskys, Mauro Fisberg, Marianella Herrera-Cuenca, Lilia Yadira Cortés Sanabria, Martha Cecilia Yépez García, Rossina Gabriella Pareja Torres, Attilio Rigotti, Viviana Guajardo, Ioná Zalcman Zimberg, Anne Chinnock, Ana Gabriela Murillo, Juan Carlos Brenes, on behalf of the ELANS Study Group

**Affiliations:** 1 Biochemistry department, School of Medicine, University of Costa Rica, San José 11501-2060, Costa Rica; 2 Departamento de Nutrição, Faculdade de Saúde Pública, Universidade de São Paulo, São Paulo 03178-200, Brazil; 3 Faculdade de Ciências Biológicas e da Saúde, Universidade São Judas Tadeu, São Paulo 03166-000, Brazil; 4 Faculty of Medicine, School of Nutrition, Pontificia Universidad Católica de Argentina, Ciudad Autónoma de Buenos Aires C1107AAZ, Argentina; 5 Committee of Nutrition and Wellbeing, International Life Science Institute (ILSI-Argentina), Buenos Aires C1059ABF, Argentina; 6 Instituto Pensi, Fundação José Egydio Setubal, Sabará Hospital Infantil, São Paulo 01239-040, Brazil; 7 Departamento de Pediatria, Escola Paulista de Medicina, Universidade Federal de São Paulo, São Paulo 04023-062, Brazil; 8 Centro de Estudios del Desarrollo, Universidad Central de Venezuela (CENDES-UCV)/Fundación Bengoa, Caracas 1010, Venezuela; 9 Departamento de Nutrición y Bioquímica, Pontificia Universidad Javeriana, Bogotá 110111, Colombia; 10 Colegio de Ciencias de la Salud, Universidad San Francisco de Quito, Quito 17-1200-841, Ecuador; 11 Instituto de Investigación Nutricional, La Molina, Lima 15026, Peru; 12 Centro de Nutrición Molecular y Enfermedades Crónicas, Departamento de Nutrición, Diabetes y Metabolismo, Escuela de Medicina, Pontificia Universidad Católica, Santiago 833-0024, Chile; 13 School of Public Health and Preventive Medicine, Monash University, Melbourne 3004, Australia; 14 School of Nutrition, University of Costa Rica, San José 11501-2060, Costa Rica; 15 Institute for Psychological Research & Neuroscience Research Center, University of Costa Rica, San José 11501-2060, Costa Rica

**Keywords:** cross-sectional study, diet quality, diet diversity, Latin America, nutrition, nutrition assessment, survey

## Abstract

This study aimed to assess diet quality score (DQS), considering healthy and unhealthy foods and nutrients, and diet diversity score (DDS) as indicators of risk of noncommunicable diseases in eight Latin American countries, and to verify the possible differences considering country, sex, age, socioeconomic, and nutritional status. A multicenter household population-based cross-sectional survey was conducted with 9218 individuals (age range 15–65 years). Sociodemographic and anthropometric data were collected. Dietary intake was measured using two non-consecutive 24-h recalls and diet quality and diversity were assessed. In the whole sample, scores were observed from 63.0% ± 9.3% to total DQS, 65.0% ± 13.6% to healthy dietary items and 60.2% ± 13.6% to unhealthy items, and 5.6 ± 1.1 out of 9 points to DDS. Women presented lower DDS compared to men (5.5 ± 1.1 vs. 5.6 ± 1.1, *p* < 0.001). Healthy DQS was higher as the socio-economic level increased, and unhealthy DQS was the opposite (*p* < 0.05). Total DQS was significantly lower only at the low socio-economic level (*p* < 0.05). Chile and Venezuela showed the lowest healthy (62.2 ± 15.2 and 61.9 ± 11.7, *p* < 0.05) and total DQS (61.4 ± 10.3, 61.2 ± 8.7, *p* < 0.05). No effects were observed when considering the age and anthropometric measurements. Promoting consumption of a diverse and high-quality diet is an essential challenge to accomplish.

## 1. Introduction

According to the Food and Agriculture Organization of the United Nations, obesity has become the greatest nutritional threat in Latin America and the Caribbean, where up to 57.0% of the population is overweight and 23.6% obese. Simultaneously, hunger and micronutrient deficiency are still present affecting 6.1% of the regional population, especially the most vulnerable groups, such as children and women of childbearing age [1,2]. Paradoxically, many overweight individuals consume a diet low in minerals and vitamins indicative of malnutrition due to micronutrients deficiency in spite of their energy-dense dietary intake [3]. A shift towards a Western diet and disparities between socioeconomic conditions and food availability can explain the coexistence of both conditions: excess body weight and undernutrition even within the same household, which represents a dual nutritional burden and a public health challenge [4].

As a result, the concept of dietary quality has emerged in nutritional epidemiology to evaluate the population’s dietary habits, their impact on human health, and the efficacy of dietary interventions [5]. Diet quality evaluates behaviors and food preferences from a determined group and involves the assessment of both quality and variety of the entire diet, enabling examination of associations between whole foods and health status. Diet quality can be measured by scoring food patterns in terms of how closely they align with national dietary guidelines and how diverse the variety of healthy choices is within core food groups [6]. Diet quality score (DQS) is directly related with the risk of noncommunicable chronic diseases (NCCDs) since a poor score has shown to increase the risk for obesity and its comorbidities, such as diabetes, cancer, and cardiovascular diseases [5,7,8,9,10]. Conversely, higher scores have been associated with better anthropometric measurements such as lower body mass index, lower waist-to-height ratio and waist circumference [8], and also with a reduction in all-cause mortality and cancer mortality [10]. Overall, these findings suggest that higher DQS reflect better dietary patterns and could be reliable indicators of nutritional status and metabolic health.

Dietary diversity score (DDS) on the other hand, is defined as the number of food items or food groups consumed over a given period, measured at the household or individual level [11]. This concept is widely recognized as a key dimension of dietary quality because it is based on the premise that consuming a variety of foods will guarantee the intake of essential nutrients and therefore, lead to better diet quality and healthier outcomes [12]. The consumption of a wide variety of foods from distinct food groups is associated with an increased likelihood of adequate nutrients intake [13], a higher concentration of antioxidant blood markers [14], and lower cardiovascular risk factors [15] and metabolic syndrome in pre-diabetes subjects [16].

In the United States, DQS and DDS can be measured using different approaches, such as Healthy Eating Index (HEI), Alternate Healthy Eating Index (AHEI), and Dietary Approaches to Stop Hypertension (DASH) score [10]. In addition, European countries also use the MedDietScore (MDS), PREDIMED Mediterranean Diet Score, (P-MDS), and Dutch Healthy Diet-Index (DHDI), among others [8]. Other countries such as Brazil and Singapore, have reported using the Healthy Diet Indicator, the Diet Quality Index, and Overall Nutritional Quality Index [17,18]. Both DQS and DDS are cost-effective, non-invasive tools to assess dietary patterns within a population and can be used to orientate public health policies, communicate risk and targeted preventive lifestyle or pharmacological interventions [9]. Few studies, however, have assessed diet quality and diet diversity in Latin America, and to our knowledge, this is the first one conducted simultaneously using exactly the same methodology among a large representative sample of the urban Latin-American population. Therefore, this study aimed to investigate the dietary quality and diversity in eight Latin American countries, and to verify the possible differences considering multiple variables within countries such as sex, age, socioeconomic status, and nutritional status.

## 2. Materials and Methods

### 2.1. Study Population

The Latin American Study on Nutrition and Health/Estudio Latino Americano de Nutrición y Salud (ELANS) is a multicentric household-based cross-sectional study, conducted from March 2014 to December 2015 in 9218 individuals aged 15–65 years, from urban areas of eight Latin American Countries: Argentina, Brazil, Chile, Colombia, Costa Rica, Ecuador, Peru, and Venezuela. This study was designed to assess dietary intake, physical activity, and its association with anthropometric profile among a representative urban population of the participating countries. More details can be seen in Fisberg et al. [19].

Recruitment of participants involved a random complex, multistage sampling, stratified by geographical location, gender, age, and socioeconomic level (SEL) with a random selection of Primary Sampling Units and Secondary Sampling Units (SSU). Households within SSU were selected through systematic randomization. Selection of respondents within a household was done using 50% of the sample next birthday, 50% last birthday, and controlling quotas for gender, age, and SEL. SEL was evaluated by a questionnaire using a country-specific format and based on legislative requirements or established local standard layouts. Only urban areas were included considering that 80%–90% of the participant countries’ population live in urban areas. The representative sample size was established with a confidence level of 95% and a sample error of 3.9% at a 5% significance level and a survey design effect of 1.75. Sample weighting was applied at each country level. Individual within identified households were selected using 50% of sample next birthday and 50% last birthday, controlling quotas for sex, age, and SEL and screening for eligibility. Pregnant and lactating women (in the first six months postpartum), individuals with major physical or mental impairments that affect food intake or physical activity, individuals outside of age range 15–65 years, adolescents without assessment or consent of a parent or legal guardian, and individuals unable to read were not included in the sample. All participants signed a written informed consent/assent before the commencement of the study. Participants’ confidentiality for the pooled data was maintained using numeric identification codes rather than names. All data transfer was done with a secure file sharing system. Western Institutional Review Board (#20140605) and ethics review boards of participating institutions approved this study, and it was registered at Clinical Trials (#NCT02226627).

### 2.2. Anthropometric Measurements

Anthropometric measurements of body weight and height and waist, hip, and neck circumferences were obtained from all participants by trained interviewers following standardized procedures. Body weight was measured with a calibrated electronic scale up to 200 kg with an accuracy of 0.1 kg after removing heavy clothing, pocket items, shoes, and socks. Height was measured with a portable stadiometer up to 205 cm with an accuracy of 0.1 cm. Circumferences were measured with an inelastic tape to the nearest 0.1 cm. Body mass index (BMI: weight (kg)/height (m^2^)) for participants under 18 years old was classified according to the cut-off criterion proposed by de Onis for World Health Organization (WHO) in 2007 [20] and for those over 18 years BMI was defined following the WHO BMI classification: underweight if BMI ≤ 18.5 kg/m^2^, normal weight if BMI > 18.5 kg/m^2^ and < 25.0 kg/m^2^, overweight if BMI ≥25.0 kg/m^2^ and obesity if BMI ≥ 30.0 kg/m^2^. Waist circumference (WC) cut-off was established at ≥102 cm for men and ≥88 cm for women [21]. Neck circumference´s cut-off for adolescents was established at >34.25 cm for men and >31.5 cm for women [22], and for adult participants >39 cm and >35 cm for men and women, respectively [23]. 

### 2.3. Data Entry and Database

Participants were visited on two opportunities on non-consecutive days spaced up to eight days with proportional distribution of weekdays and weekends. Dietary assessment was conducted by trained interviewers applying a face-to-face 24 h dietary recall following the United States Department of Agriculture´s five-step multiple-pass method [24]. This procedure facilitated recalling of all foods, non-alcoholic and alcoholic beverages, water, recipes, and dietary supplements consumed over the 24 h prior to the interview. This method included the following steps: (1) Quick list: the interviewees were asked to list all foods and beverages consumed the previous day without interruption. (2) Forgotten foods: the interviewer repeated the list of foods and beverages mentioned by the interviewee to identify foods that could have been forgotten. (3) Time and occasion of consumption are included. (4) Detail cycle: the interviewer asked for details on descriptions and amounts of each food reported; each occasion and intervals between occasions were also reviewed. (5) Final review probe: the interviewer repeated all information to collect data of additional foods not remembered earlier. Food servings were estimated using photographic albums containing the most commonly consumed foods and household utensils standardized for each country. A total of 18,436 24-h recalls—two for participant—were obtained. Trained nutritionists supervised data collection and were responsible for converting the measures obtained into grams and milliliters. Data collected were analyzed using the Nutrition Data System for Research software version 2014 (NDS-R)—a dietary assessment tool developed by the Nutrition Coordinating Center of Minnesota, Minneapolis, MN, based on the US Department of Agriculture composition table. Before entering data on the NSDS-R software, professional nutritionists in each country followed a standardization procedure for matching local foods to US Department of Agriculture foods [25]. A total of 18,436 24-h dietary recalls were assessed to obtain the age-specific, sex-specific, and nation-specific usual intake of the 17 selected food groups and nutrients, which were classified in quintiles and assigned the correspondent scoring.

### 2.4. Diet Quality

There are many different options for assessing diet quality such as Healthy Eating Index (HEI), Alternate Healthy Eating Index (AHEI), and Dietary Approaches to Stop Hypertension (DASH) score described elsewhere [6]. To develop the diet quality score, we followed the methodology used by Imamura et al. [26]. This proposal outstrips those approaches as it has the following advantages: it is based on actual food consumption, it allows assessing the intake of both desirable and detrimental food groups and nutrients independently. This approach evaluated consumption of key dietary items, adjusted for a 2000 kcal per day diet and modeling two different dietary patterns: one based on the relatively high consumption of 10 healthy items (e.g., fruits, vegetables, beans and legumes, nuts and seeds, whole grains, milk, total polyunsaturated fatty acids, fish, plant omega-3s, and dietary fiber) and a second one based on the relatively low consumption of seven unhealthy items (e.g., unprocessed read meats, processed meats, sugar-sweetened beverages, saturated fat, trans fat, dietary cholesterol, and sodium). A third pattern incorporated all dietary factors together. Items were selected due to the probable or convincing evidence of having casual effects on major NCCDs, including cardiovascular diseases, diabetes, and diet-related cancers, including protective or harmful effects [27,28]. To obtain the score for each pattern, the usual intake of each dietary factor was divided into age-specific, sex-specific, and country-specific quintiles, across 64 subgroups, including men and women from four age categories and eight countries. An ordinal score (1 to 5) was assigned to each quintile, given the highest score (5) to the highest mean intake of healthy items, and the lowest mean intake of unhealthy items. Having 10 healthy items, the highest dietary quality score (DQS) for the healthy pattern was 50 points and 35 points for the unhealthy pattern, which has seven items. These DQS were summed to obtain the overall diet quality score of 85 points. All DQS were standardized to a 100-point scale and the higher the scores the higher the healthy or unhealthy diet.

### 2.5. Diet Diversity Score

Dietary diversity score (DDS) was assessed following the Food and Agriculture Organization’s guidelines for measuring household and individual dietary diversity [11]. DDS was calculated at the individual level, based on the Women’s Dietary Diversity Score Project [29] food groups classification, that included the first as the sum of numbers of food groups reported to be consumed over the day before to the first 24-h recall. The following nine food groups were used: (1) cereals, (2) white roots and tubers, (3) vegetables; (4) fruits; (5) meat, poultry, offal; (6) fish and seafood; (7) eggs; (8) pulses, legumes, and nuts; and (9) milk and milk products. The consumption of at least 15 g of each food group was assigned one point (if consumed) or zero points (if consumption was less than 15 g). A total of nine points could be obtained to the maximum variability diet. Higher scores indicated higher dietary diversity as more food groups were eaten.

### 2.6. Statistical Analyses

To evaluate diet quality, the usual dietary intake of each nutrient or grams of food group was estimated using the Multiple Source Method—a web-based tool developed by researchers of the European Prospective Investigation into Cancer and Nutrition (EPIC) available at http://msm.dife.de/. This tool estimates usual dietary intake of nutrients and foods consumed by populations and individuals. Usual intake of nutrients (g, mg or μg) and food groups intake (g) were presented as means and standard deviation and were stratified by percentiles and country. All DQS and DDS were also presented as mean and standard deviation, stratified by gender, age group, SEL, country, nutritional status, waist, and neck circumference classification. Subjects were categorized based on DDS tertiles cut points: 1st (0 to 4 points), 2nd, (>4 to <6 points) and 3rd (higher than 6 points). Data were compared using the multivariate variance analysis (MANOVA). Groups of foods and nutrients, the three DQS and the DDS, quintiles of DQS and tertiles of DDS were the dependent variables. The between-groups factors were all the sociodemographic and anthropometric variables. When appropriate, multiple comparisons were performed following the Bonferroni post-hoc test. The effect size was estimated by partial eta-squared coefficients (ƞ^2^_p_). Pearson correlations (*r*) and multiple linear regression analysis (*R* and *R^2^*) were computed with the dependent variables in a multilevel fashion within selected groups of sociodemographic and anthropometric variables. Statistical significance was defined as *p* < 0.05.

## 3. Results

### 3.1. Usual Intake of Selected Food Groups or Nutrients

The study sample included 9218 participants, 52.2% women, and a mean age of 35.8 years. Table 1 summarizes the mean usual intake of food groups and nutrients of dietary factors contributing to the dietary patterns for the whole sample and for each of the eight participant countries. As expected, intake of healthy and unhealthy foods varied among all quintiles (all *p*-values < 0.0001) with higher intake observed in the fifth quintile (Bonferroni, all *p*-values < 0.0001). Table 1 shows food items ranked from the largest to the smallest ratio between the fifth and the first quintile. The largest differences were observed within the healthy dietary factors, with the usual consumption of nuts and seeds being 197.2-fold higher between the fifth and the first quintile. The lowest difference between quintiles yielded a ratio of 1.92 observed for the percentage of energy obtained from polyunsaturated fat. The variability within the unhealthy dietary factors was substantially smaller with a difference between the fifth and the first quintile ranging from 7.24 to 1.73 for the intake of processed meats and sodium, respectively.

Table 2 summarizes the mean and standard deviation of usual intake adjusted for 2000 kcal per day of the 17 dietary factors contributing to the dietary patterns for the whole sample and for each of the eight participant countries. Dietary factors were ranked according to their size effect (i.e., partial eta-squared coefficient), starting from the largest effects observed among countries, as follows. From the healthy foods/nutrients, the highest variation in usual intake of food groups among countries was noted for beans and legumes (*p* = 0.0001; ƞ^2^_p_ = 0.453), with Costa Rica showing a consumption (103.64 ± 56.40 g/d) 41-fold higher than that reported in Argentina (2.52 ± 7.79 g/d). Brazil was the second highest consumer of beans and legumes (59.34 ± 34.28 g/d), followed by Ecuador (47.95 ± 28.81 g/d). For omega-3 fats obtained from plants (*p* = 0.0001; ƞ^2^_p_ = 0.336), Costa Rica (0.18 ± 0.09 g/d) reported the highest intake, which differed from that observed in the rest of the countries (Bonferroni, *p* < 0.05). In second place, Ecuador and Peru had exactly the same intake of omega-3 fats (0.12 ± 0.02 g/d), which was above the consumption of all other nations (Bonferroni, *p* < 0.05). Usual intake of dietary fiber (*p* = 0.0001; ƞ^2^_p_ = 0.263) was comparable among countries with the exception of Costa Rica (21.98 ± 6.44 g/d), which showed an intake above all other nations (Bonferroni, *p* < 0.05). In contrast, Argentina had the lowest dietary fiber intake (10.89 ± 3.36 g/d) (Bonferroni, *p* < 0.05). The consumption of vegetables (*p* = 0.0001; ƞ^2^_p_ = 0.210) in Chile (171.68 ± 76.03 g/d), Ecuador (163.12 ± 61.53 g/d), and Costa Rica (146.88 ± 77.02 g/d) was higher than that reported in all other countries (Bonferroni, *p* < 0.05). Colombia (89.57 ± 43.19 g/d) and Brazil (88.69 ± 70.17 g/d) showed a lower intake of vegetables than in the rest of the countries (Bonferroni, *p* < 0.05). The usual intake of milk (*p* = 0.0001; ƞ^2^_p_ = 0.140) reported in Colombia (172.96 ± 108.55 g/d) and Brazil (123.09 ± 120.26 g/d) was higher than that in all other countries (Bonferroni, *p* < 0.05). The third highest consumption was observed in Ecuador (96.85 ± 67.73 g/d) and the lowest in Peru (42.16 ± 31.26 g/d). Consumption of fruits (*p* = 0.0001; ƞ^2^_p_ = 0.102) was particularly high in Chile (123.25 ± 89.66 g/d) and Peru (116.74 ± 85.40 g/d) (Bonferroni, *p* < 0.05). The rest of the countries exhibited a comparable consumption, with the exception of Venezuela that showed a rather low fruit intake (27.30 ± 51.43 g/d). The percentage of energy obtained from polyunsaturated fatty acids (*p* = 0.0001; ƞ^2^_p_ = 0.098) was higher in Ecuador (9.12% ± 1.81%) as compared to the rest of the countries (Bonferroni, *p* < 0.05), whereas Chile had a percentage below that observed in all other nations (6.67% ± 1.70%). For the rest of the countries, the percentage of energy derived from polyunsaturated fatty acids remained within a strict range of 1%. The usual consumption of fish (*p* = 0.0001; ƞ^2^_p_ = 0.097) was equally higher in Peru (28.46 ± 21.05 g/d) and Ecuador (28.32 ± 20.27 g/d), which differed in their intake compared with all other countries (Bonferroni, *p* < 0.05). In a subsequent level, Brazil (23.07 ± 29.36 g/d) and Costa Rica (22.50 ± 14.78 g/d) showed higher fish consumption than that in the remaining countries (Bonferroni, *p* < 0.05), whereas Argentina exhibited the lowest intake in the region (6.45 ± 14.23 g/d). For nuts and seeds (*p* = 0.0001; ƞ^2^_p_ = 0.031), Colombia (4.36 ± 16.28 g/d) and Ecuador (3.70 ± 6.43 g/d) showed the highest consumption (Bonferroni, *p* < 0.05), whereas Venezuela (0.47 ± 3.87 g/d), Argentina (0.82 ± 2.84 g/d), and Chile (0.93 ± 4.38 g/d) showed the lowest usual consumption of these foods (Bonferroni, *p* < 0.05). Regarding wholegrain consumption (*p* = 0.0001; ƞ^2^_p_ = 0.019), Costa Rica (14.32 ± 17.32 g/d) exhibited a consumption higher than level of the other countries, except Peru (12.29 ± 13.36 g/d) (Bonferroni, *p* < 0.05), which had the second highest consumption followed by Ecuador (10.48 ± 17.54 g/d). In contrast, Venezuela (6.06 ± 10.29 g/d) and Brazil (7.24 ± 21.10 g/d) showed the lowest wholegrain intake (Bonferroni, *p* < 0.05), which was different from that observed in all countries, except Chile (8.02 ± 22.25 g/d) that showed the third lowest consumption. 

For the unhealthy items, the largest difference among countries was found in the usual intake of sodium (*p* = 0.0001; ƞ^2^_p_ = 0.713) (0.5% ± 0.2%), with Ecuador (4.52 ± 0.77 g/d) reporting a consumption 4.6-fold higher than that in Peru (0.97 ± 0.24 g/d), which showed the lowest intake. Consumption in both countries differed from that observed in the rest of the nations (Bonferroni, *p* < 0.05). The usual percentage of energy derived from trans fats ( *p*= 0.0001; ƞ^2^_p_ = 0.344) (0.5% ± 0.2%) was higher in Brazil (1.33% ± 0.64%) followed by Argentina (1.00% ± 0.24%), Chile (1.00% ± 0.28%), and Colombia (1% ± 0.26%), which had quite comparable percentages that also differed when compared to those in the rest of the countries (Bonferroni, *p* < 0.05) except Venezuela (0.96% ± 0.25%). Peru, in contrast, had the lowest percentage of energy obtained from trans fats (0.49% ± 0.18%). The usual percentage of energy derived from saturated fat (*p* = 0.0001; ƞ^2^_p_ = 0.323) was higher in Argentina (11.70% ± 2.38%), Chile (10.80% ± 2.29%), and Colombia (10.62% ± 2.13%) than in the other countries (Bonferroni, *p* < 0.05). In a second level appeared Venezuela (9.87% ± 2.02%) and Brazil (9.77% ± 2.32%), with percentages above those in the other countries (Bonferroni, *p* < 0.05). On the opposite extreme, Peru (6.49% ± 1.46%) reported again the lowest percentage of energy derived from saturated fat. The usual consumption of unprocessed red meat (*p* = 0.0001; ƞ^2^_p_ = 0.284) was higher in Brazil (94.71 ± 43.48 g/d) and Argentina (78.62 ± 34.26 g/d), which differed from that in all other countries (Bonferroni, *p* < 0.05). Colombia (71.07 ± 29.29 g/d) reported the third highest intake, whereas Peru (29.30 ± 20.27 g/d) had the lowest consumption of unprocessed red meat. The usual consumption of sugar-sweetened beverages (*p* = 0.0001; ƞ^2^_p_ = 0.272) was higher in Argentina (1092.90 ± 650.31 g/d) followed closely by that in Peru (920.17 ± 325.93 g/d). Intake in both countries differed from that in all other nations (Bonferroni, *p* < 0.05). At the other end of the spectrum was Chile (331.73 ± 240.66 g/d), with the lowest usual intake of sugar-sweetened beverages. For usual consumption of processed red meat (*p* = 0.0001; ƞ^2^_p_ = 0.176), Chile (29.77 ± 21.70 g/d), and Brazil (26.65 ± 20.49 g/d) reported the highest values, which were above those in the rest of the countries (Bonferroni, *p* < 0.05). In a subsequent level, Costa Rica and Argentina showed a similar consumption, which was higher than that observed in the remaining countries (Bonferroni, *p* < 0.05). Peru, on the other hand, had the lowest intake of processed red meat (6.72 ± 3.90 g/d). For the mean usual intake of cholesterol (*p* = 0.0001; ƞ^2^_p_ = 0.113), Argentina (346.85 ± 90.67 mg/d) and Costa Rica (247.71 ± 97.14 mg/d) showed the highest and lowest values, respectively; which differed as compared with intake in the other countries (Bonferroni, *p* < 0.05). The second highest cholesterol intake was found in Colombia (329.61 ± 95.06 mg/d), which was higher than that reported in the remaining nations (Bonferroni, *p* < 0.05).

### 3.2. Diet Quality and Diet Diversity Scores

The mean total diet quality score (DQS) was of 63.01% ± 9.21% including all countries. For the healthy and the unhealthy DQSs, a mean of 64.96% ± 13.61% and 60.22% ± 13.63% were obtained, respectively (see Table 3). In the whole sample, the mean diet diversity score (DDS) was 5.58 ± 1.13, ranging from 0 to 9 points. When comparing all DQSs and the DDS among sociodemographic variables, no differences were detected for sex or age group (N.S), except for the DDS (*p* = 0.03; ƞ^2^_p_ = 0.001), which was lower in women. A higher percentage of men consuming starchy staples, eggs, nuts and legumes, meat and fish, organs and other fruit and vegetable groups was observed, while a higher percentage of women consuming dark green vegetables, milk, and vitamin A rich fruits and vegetable groups. The DQSs and DDS differed according to the SEL (healthy: *p* = 0.0001; ƞ^2^_p_ = 0.003; unhealthy: *p* = 0.002; ƞ^2^_p_ = 0.002; total: *p* = 0.03; ƞ^2^_p_ = 0.001). Specifically, the healthy DQS and DDS were higher as the SEL increases, whereas the unhealthy DQS showed the opposite pattern being higher in the middle and lower SEL (Bonferroni, *p* < 0.05). The total DQS was lower only at the low SEL (Bonferroni, *p* < 0.05). (Dietary patterns by country, according to socioeconomic are shown in Table S1.) When comparing the countries, the DDS did not vary among them. However, the healthy DQS (*p* = 0.0001; ƞ^2^_p_ = 0.005) and total DQS (*p* = 0.001; ƞ^2^_p_ = 0.001) did differ, with Chile and Venezuela showing the lowest scores as compared with the rest of the countries (Bonferroni, *p* < 0.05), which varied among each other in less than 1%. None of the scores were different among all anthropometric measurements (e.g., waist or neck circumference and nutritional status).

In terms of explained variance, the contribution of the healthy DQS to the total DQS was more than twice the value of the unhealthy DQS (*R^2^* = 0.64 vs. and *R^2^* = 0.26, respectively) (see Table S2). The DDS was predicted only by the healthy DQS in 17% (*R* = 0.171, *p* = 0.0001). For sex, such a prediction was slightly higher in men (*R* = 0.184, *p* = 0.0001) than in women (*R* = 0.158, *p* = 0.0001). Regarding age, the relationship between DDS and healthy DQS was significant at every age interval (range of *R* coefficients = 0.162–0.175, all *p*-values < 0.0001) (see Table S2). The relationship between the healthy DQS and the DDS was higher in the middle and high SEL than in the low level (range of *R* coefficients = 0.155–0.183, all *p*-values < 0.0001) (see Table S2). Among countries, the relationship between the healthy DQS and the DDS varied substantially from 8% to 29% in Ecuador and Brazil, respectively (range of *R* coefficients = 0.086–0.287, all *p*-values < 0.0001) (see Table S2). Among overweight subjects (*R* = 0.159, *p* = 0.0001) and wider waist circumference (*R* = 0.129, *p* = 0.0001), the relationship between healthy DQS and the DDS was lower than in subjects with normal weights (*R* = 0.189, *p* = 0.0001) and narrower waistlines (*R* = 0.190, *p* = 0.0001) (see Table S2).

**Figure 1:**
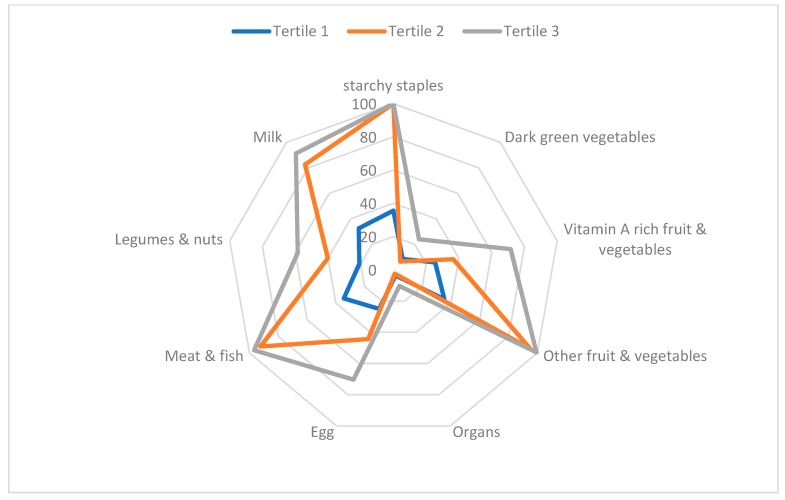
Frequency of consumption for the nine food groups that determinate diet diversity score according to tertile of scores.

When dividing the DDS in tertiles, it was found that all DQSs differed among tertiles (all *p*-values < 0.0001). The largest differences were detected for healthy DQS, which ranged from 63.0% to 68.5% (Bonferroni, *p* < 0.05). In addition, the food groups were differentially distributed among tertiles, as shown in Figure 1. The more frequently reported dietary group was starchy staples—consumed by 99.5% of the whole sample. Next, there were groups comprising the meat/fish and the fruit/vegetables, which were consumed by 85.4% and 82.2% of participants, respectively. The least consume foods were the dark green vegetables and organs meat, consumed only by 6.9% and 2.9% of participants, respectively (Figure 1).

## 4. Discussion

The present study provides insight into dietary quality and dietary diversity among Latin Americans living in urban areas, showing that consumptions of healthy and high diversity diets differ among the eight countries participating in the study. The relationship between dietary factors and major causes of morbidity and mortality have been extensively studied worldwide [7,30,31]. By considering food groups and nutrients that are relevant for their effects on the risk of NCCDs, we provided a comprehensive picture of food consumption in Latin America and its potential effect on health outcomes.

A recent analysis of the Global Burden of Disease, Injuries, and Risk Factors Study (GBD) 2017 [30], pointed out the potential impact of suboptimal diet on NCCDs. There it was highlighted the likely effect of a diet low in fruits, vegetables, and whole grains and high in sodium, that could be related to two-thirds of diet-related to disability-adjusted life years. In the present study, it was observed an usual daily intake of fruits, vegetables, and whole grains far below the level of intake required to minimize the risk from all causes of death suggested by GBD: 80.76 g/d vs. 200–300 g/d for fruits, 113.17g/d vs. 290–430 g/d of vegetables and 9.32g/d vs. 100–150 g/d of whole grains, and a higher consumption of processed meat and sugar-sweetened beverages: 20.44 g/d vs. 0–4 g/d of processed read meats and 683.12 g/d vs. 0–5 g/d of sugar-sweetened beverages. Regarding sodium intake, we estimate a mean usual intake of 2.69 g/d, which is higher than recommendation of <2.0 g/d [32]. It is worth noting that sodium intake in this analysis may have been underestimated, due to the inaccuracy to calculate sodium contained in processed food or the amount added while cooking or eating. The dietary pattern previously described is reflected in the results of diet quality and diet diversity analysis.

Overall diet quality was higher than reported by Imamura [26] who reported a global dietary pattern score in 2010 of 44.0 ± 10.5 based on 10 healthy items, 52.1 ± 18.6 based on seven unhealthy items, and 51.9 ± 9.3 based on all 17 items evaluated. In our population the mean scores were 64.9 ± 13.6, 60.2 ± 23.6 and 63.0 ± 9.21 for score based on 10 healthy items, seven unhealthy items, and all 17 items, respectively in 2014–2015. Nevertheless, it has to be taken into consideration that our analysis includes only the urban population of the eight countries involved in the study, and differences could be observed if rural regions were included in the analysis.

Compared with similar analyses in other developing countries, that used nine food groups to build the diet diversity score, the mean DDS in overall ELANS was higher (5.78 ± 1.1 for the whole sample and 5.51 ± 1.1 for woman) than that reported by Savy et al. (2008) [33], which was of 4.9 in women of urban Burkina Faso, 4.7 ± 1.5 reported by Narmaki et al. (2015) [14] in women of a Tehran municipality, and 4.43 and 4.9 for cases and controls, respectively, reported in a study conducted by Gholizadeh et al. (2018) [16], among pre-diabetes male and female subjects. Our results showed that, while there are no significant differences in diet quality by sex, there are differences in terms of diet diversity, with males having a significantly more diverse diet than women (Table 3). Certainly, 52.5% of men accomplish the recommendation of including at least five groups in the diet, whereas only 48.5% of women did. Even though women are reporting a less diversified diet, they tend to include more nutrient density food such as dark green vegetables, milk, and vitamin A rich fruits and vegetables than men, which could clearly contribute to better accomplish of micronutrient recommendations. Differences among sexes could be explained by social factors that go beyond biological differences such as gender roles, work patterns, level of education, nutritional knowledge, and food choices.

It has been described that differences in diet quality when analyzed by age, may be due to different social characteristics. For example, social contact tends to decrease at older age, and it could be one of the causes of a poor diet quality found in older adults. Marriage can also be associated with better food choices, and peer pressure in adolescents is a high determinant in dietary patterns in this age group [34,35]. Conversely, there were no differences in diet quality by age group. However, we did find a tendency to a higher DDS in older than in younger participants.

It can be expected that cultural and culinary differences may account for strong determinants in diet quality and diet diversity. However, it has been reported that dietary patterns do not change that much across Latin America [36], and in this study there were no notable differences in DDS by country. We found that both Chile and Venezuela have a significant lower score for healthy food items and the total DQS, but they did not differ from the other countries in the unhealthy score. Particularly, in Venezuela, low consumption of fruits and vegetables have been reported since 2014, when food insecurity of households due to lack of income enough to buy foods started to be a factor for lowering the quality of the diet, altering the food pattern, and reducing the amount of foods consumed by families [37,38]. A study performed by Pinto et al [39], in the Chilean urban population from ELANS study, reported diet quality to be far from optimal when assessed through the application of the Alternate Healthy Eating Index 2010. A greater heterogeneity was observed for DDS, where Brazil showed a significantly lower score compared with other countries and Ecuador a significantly higher one. The differences observed among countries regarding DQS, but not DDS, can be explained by inherent characteristics of each scoring procedure. The DDS does not account for the type of carbohydrates such as whole or refined grains, added and total sugars, they are all score positively. The same occurred for sources of animal proteins, that are positively scored in DDS, but includes red meat and saturated fats which have been associated with the risk of chronic diseases. Therefore, as DDS does not adequately distinguish between healthy and unhealthy food items as DQS does, it may not be appropriate enough to point out the differences among countries.

Our study suggests that socioeconomic status is a strong determinant factor for diet quality and diet diversity (Table 3). There were significant differences between high, middle, and low income when diet quality is measured by healthy food consumption, the low-income group being the one with the lowest score. However, when diet quality was measured by the fewer unhealthy items consumption, the difference between low and middle income disappears, and the upper class appears to be the one with the lower score, which means that by both measurements the high SEL has a better diet quality. This pattern has been widely described by other authors [40,41,42,43]. Nevertheless, it is important to highlight that Peru had a significantly higher total diet quality score in the low SEL than in high SEL, attributable to a significantly lower intake of unhealthy food items. For the whole sample, also participants in the more privileged socioeconomic position were those with higher DDS. In a study conducted with adults in Australia, Livingstone et al. showed that individuals with lower SEL have poorer diets when compared to those with higher SEL [43]. This could be due to the fact that socially disadvantaged groups have a more caloric-dense—but nutrient poor—diet, with lower intake of fruits and vegetables [44]. This type of food is more likely to be consumed in higher quantity and variety by people in higher SEL quintiles [38]. One explanation for this dietary pattern is that healthy items have a higher economic cost, which makes them accessible to only a fraction of the population [41,45].

Unlike previous studies that have found a relationship between DQS or DDS and nutritional status [46,47,48,49,50,51], we did not observe this association. Significant inverse association of several DQSs with BMI and WC have been reported [47,49,51], Asghari et al. [52] documented no significant association. Studies aimed to explore the association between DDS and obesity have yielded controversial results. While some studies have observed a direct association among DDS and obesity [53,54], others have shown that a higher DDS was associated with a healthier diet and lower BMI [51,55]. Karembeike et al. [53] reported that obese Iranian adults showed higher DDS than overweight and normal-weight participants, consistent with other studies reporting that adults with higher DDS had higher energy intake [51]. A higher proportion of total energy intake from total and saturated fat is linked to obesity [54]. On the other hand, a higher DDS has also been associated with higher fruit and vegetables consumption, which could lead to a reduced risk of obesity [54]. A systematic review and meta-analysis conducted by Salehi-Abargouei [56], showed no significant association between DDS and BMI status, which may be attributed to the use of different methods for assessing dietary intake and estimation of the DDS. We suggest that this lack of association can also be explained by the fact that this score does not consider the quantity of food consumed, nor the physical activity levels or exercise’s energy expenditure as counterbalance of energy intake.

This study had several strengths. The ELANS has a large sample size from a nationally representative population of eight Latin American countries. Participants were disease free at the time of data collection, which reduced the possibility of disease-related recall bias. The use of two non-consecutive R-24, including weekdays and weekends, and the use of usual food intake to evaluate food groups and nutrients consumption instead of mean approaches provides more accurate information. Bias due to misreporting of energy intake that were previously assessed in this population [57], were also minimized when dietary intakes were adjusted for a 2000 kcal per day. Regarding diet quality score, analyzing dietary patterns by greater consumption of healthy items and lesser consumption of unhealthy items allowed a more comprehensive analysis of the two dietary patterns separately. Using a data-driven approach to evaluate dietary quality sheds light into the actual food intake of the population. At the same time, using this approach could also be a limitation of the analysis. For instance, if the overall consumption of a given healthy food item is low for the whole sample, it could have positioned a participant into a high quintile and receiving a high score even when that quantity is lower than desirable. So higher scores do not necessarily reflect an adequate nutrient intake when comparing to requirements or food groups according to quantities or servings recommendations. Finally, our data analysis is limited to urban populations, and these results should not be extrapolated to rural areas or to other countries of Central or South America. On the other hand, this methodology does not include moderate levels of alcohol intake as a positive component of the healthy score or high levels of alcohol as an unhealthy component. Alcohol consumption might be included in future research, considering the high prevalence of alcohol use disorders in Latin America [58].

## 5. Conclusions

There is a lower overall diet quality score in Chile and Venezuela and individuals of low SEL. Low dietary diversity was found in all studied countries and the main concern is not only the limited consumption of diverse food groups but the low frequency of consumption of micronutrients-rich food groups, such as fruits and vegetables rich in vitamin A, dark green leafy vegetables, legumes, and nuts. In such a context, promoting consumption of a diverse and high-quality diet geared towards achieving those requirements represents an important challenge for the region.

## Figures and Tables

**Table 1:** Dietary consumption of selected foods groups and nutrients in individuals residing in urban areas of Latin American countries (*n* = 9218) according to quintiles.

Food/Nutrients	Quintil 1	Quintil 2	Quintil 3	Quintil 4	Quintil 5	Quintil 5/Quintil 1 Ratio
	*n* = 1870	*n* = 1841	*n* = 1847	*n* = 1841	*n* = 1819	
Healthy items ****						
Nuts and seeds (g/d)	0.05 (0.11)	0.29 (0.29)	0.57 (0.55)	1.21 (1.15)	9.86 (16.49)	197.20
Wholegrain (g/d)	0.95 (0.84)	1.64 (1.42)	2.87 (2.97)	6.87 (7.60)	34.74 (24.81)	36.57
Fruits (g/d)	15.57 (11.95)	31.74 (22.32)	56.61 (35.22)	100.75 (48.44)	201.73 (92.27)	12.96
Vegetables (g/d)	53.75 (26.87)	81.81 (30.49)	105.14 (34.57)	132.10 (39.64)	195.00 (74.61)	12.92
Fish (g/d)	5.37 (3.00)	8.65 (4.24)	12.66 (6.86)	20.49 (11.79)	51.63 (29.12)	9.61
Beans and legumes (g/d)	13.76 (11.78)	24.16 (19.98)	33.62 (27.02)	47.70 (35.29)	81.03 (52.87)	5.89
Milk (g/d)	18.69 (15.22)	40.29 (27.88)	72.65 (41.21)	125.29 (58.73)	241.52 (115.09)	3.63
Polyunsaturated fat (% energy)	5.31 (0.88)	6.57 (0.68)	7.44 (0.70)	8.39 (0.77)	10.19 (1.45)	2.24
Dietary fiber (g/d)	11.20 (2.61)	13.94 (2.72)	15.86 (3.13)	18.12 (3.54)	22.82 (5.23)	2.04
Plant omega-3 fat (g/d)	0.05 (0.02)	0.07 (0.03)	0.09 (0.04)	0.12 (0.05)	0.17 (0.07)	1.92
Unhealthy items ****						
Sugar-sweetened beverages (g/d)	268.08 (153.27)	492.77 (187.07)	656.20 (229.80)	832.97 (287.5)	1178.17 (487.43)	7.24
Unprocessed red meats (g/d)	30.34 (12.53)	48.82 (17.40)	64.83 (20.92)	82.20 (24.52)	114.13 (37.15)	4.39
Processed meats (g/d)	5.97 (2.56)	10.64 (3.97)	17.14 (6.86)	25.80 (10.08)	43.20 (19.26)	3.76
Saturated fat (% energy)	6.92 (1.49)	8.58 (1.42)	9.65 (1.57)	10.75 (1.70)	12.68 (2.21)	2.48
Trans fat (% energy)	0.58 (0.17)	0.77 (0.19)	0.91 (0.24)	1.08 (0.32)	1.44 (0.60)	2.25
Cholesterol (mg/d)	186.10 (42.83)	242.35 (33.38)	280.71 (35.99)	324.12 (40.92)	419.64 (84.14)	1.83
Sodium (g/d)	2.00 (0.75)	2.41 (0.83)	2.66 (0.88)	2.92 (0.94)	3.45 (1.13)	1.73

Data are the mean usual intake adjusted for a 2000 kcal per day by quintile (standard deviation). g/d: grams per day. Quintiles were estimated by age-specific, sex-specific, and country-specific subgroup of subjects. **** Intake of all healthy and unhealthy foods/nutrients varied among quintiles, *p* < 0.0001 (see text for details).

**Table 2:** Dietary consumption of foods groups and nutrients in individuals residing in urban areas of Latin American countries (*n* = 9218).

Food/Nutrients	All Countries (*n* = 9218)	Countries							
		Argentina (*n* = 1266)	Brazil (*n* = 2000)	Chile (*n* = 879)	Colombia (*n* = 1230)	Costa Rica (*n* = 798)	Ecuador (*n* = 800)	Peru (*n* = 1113)	Venezuela (*n* = 1132)
Healthy items ****									
Beans and legumes (g/d)	39.87 (39.95)	2.52 (7.79)	59.34 (34.28)	21.81 (29.28)	41.61 (24.13)	103.64 (56.40)	47.95 (28.81)	23.53 (17.79)	24.81 (23.04)
Plant omega-3 fat (g/d)	0.10 (0.06)	0.32 (0.20)	0.10 (0.05)	0.11 (0.06)	0.09 (0.04)	0.18 (0.09)	0.12 (0.05)	0.12 (0.05)	0.10 (0.05)
Dietary fiber (g/d)	16.36 (5.31)	10.90 (3.36)	15.57 (4.65)	16.88 (4.78)	17.31 (4.27)	21.98 (6.44)	17.21 (3.83)	17.73 (4.60)	16.48 (4.51)
Vegetables (g/d)	113.17 (65.59)	100.83 (49.52)	88.69 (70.17)	171.68 (76.03)	89.57 (43.19)	146.88 (77.02)	163.12 (61.53)	107.76 (36.06)	96.68 (40.04)
Milk (g/d)	99.08 (100.63)	73.54 (80.59)	123.09 (120.26)	93.84 (114.39)	172.96 (108.55)	86.03 (94.37)	96.85 (67.73)	42.16 (31.26)	75.72 (68.52)
Fruits (g/d)	80.76 (83.38)	75.02 (77.20)	83.81 (86.85)	123.25 (89.66)	66.95 (70.25)	79.92 (86.93)	83.25 (76.25)	116.74 (85.40)	27.30 (51.43)
Polyunsaturated fat (% energy)	7.59 (1.90)	7.92 (2.16)	7.34 (1.92)	6.67 (1.70)	7.24 (1.48)	7.83 (1.67)	9.12 (1.81)	7.21 (1.44)	7.71 (1.98)
Fish (g/d)	19.63 (22.07)	6.45 (14.23)	23.07 (29.36)	15.71 (16.20)	15.11 (18.33)	22.50 (14.78)	28.32 (20.27)	28.46 (21.05)	19.44 (19.69)
Nuts and seeds (g/d)	1.95 (7.54)	0.82 (2.84)	1.22 (4.34)	0.93 (4.38)	4.36 (16.28)	2.20 (7.74)	3.70 (6.43)	2.74 (4.38)	0.47 (3.87)
Wholegrain (g/d)	9.32 (1.28)	9.87 (16.99)	7.24 (21.10)	8.02 (22.25)	9.38 (12.94)	14.32 (17.32)	10.48 (17.54)	12.29 (13.36)	6.06 (10.29)
Unhealthy items ****									
Sodium (g/d)	2.69 (1.04)	2.60 (0.50)	2.96 (0.64)	2.93 (0.58)	1.95 (0.51)	3.02 (0.56)	4.52 (0.77)	0.97 (0.24)	3.1 (0.52)
Trans fat (% energy)	0.95 (0.45)	1.00 (0.24)	1.33 (0.64)	1.00 (0.28)	1.00 (0.26)	0.71 (0.23)	0.70 (0.18)	0.49 (0.18)	0.96 (0.25)
Saturated fat (% energy)	9.70 (2.58)	11.70 (2.38)	9.77 (2.32)	10.80 (2.29)	10.62 (2.13)	8.74 (2.09)	8.95 (1.93)	6.49 (1.46)	9.87 (2.02)
Unprocessed red meats (g/d)	67.83 (37.36)	78.62 (34.26)	94.71 (43.48)	57.42 (29.23)	71.07 (29.29)	49.39 (21.42)	62.17 (25.67)	29.30 (20.27)	67.72 (26.56)
Sugar-sweetened beverages (g/d)	683.13 (425.29)	1092.90 (650.31)	611.65 (323.11)	331.73 (240.66)	482.99 (227.95)	702.40 (351.13)	677.22 (234.74)	920.17 (325.93)	597.92 (290.73)
Processed meats (g/d)	20.44 (16.71)	22.39 (12.36)	26.65 (20.49)	29.77 (21.70)	15.51 (11.79)	23.40 (15.15)	11.29 (9.98)	6.72 (3.90)	21.14 (14.08)
Cholesterol (mg/d)	289.94 (93.78)	346.85 (90.67)	278.40 (89.48)	285.00 (100.98)	329.61 (95.06)	247.71 (97.14)	257.37 (69.27)	279.74 (75.04)	270.26 (83.24)

Data are the mean usual intake adjusted for a 2000 kcal per day. g/d: grams per day. See main text for details about the differences of dietary scores among sociodemographic and anthropometric variables. **** Intake of all healthy and unhealthy foods/nutrients varied among countries, *p* < 0.0001 (see text for details).

**Table 3:** Global dietary patterns in individuals residing in urban areas of Latin American countries, according to country, sex, age groups, socioeconomic level, and weight status.

		Score Based on Greater Consumption of 10 Healthy Dietary Items	Score Based on Lesser Consumption of Seven Unhealthy Items	Score Based on 17 Dietary Items	Diet Diversity Score
Variables	*n* (%)	Mean (SD)	Mean (SD)	Mean (SD)	Mean (SD)
Total countries	9218 (100)	64.96 (13.61)	60.22 (13.62)	63.01 (9.29)	5.58 (1.13)
Sex					
Male	4409 (47.83)	64.91 (13.68)	60.23 (13.91)	62.98 (9.24)	5.67 (1.13)
Female	4809 (52.17)	65.00 (13.70)	60.22 (13.36)	63.03 (9.34)	5.51 (1.13) ***
Age group					
15–19 years	1223 (13.27)	64.77 (13.16)	60.36 (13.71)	62.96 (9.03)	5.48 (1.19)
20–34 years	3479 (37.74)	65.00 (13.80)	60.17 (13.63)	63.01 (9.24)	5.56. (1.13)
35–49 years	2627 (28.50)	64.98 (13.96)	60.20 (13.59)	63.01 (9.24)	5.65 (1.11)
50–65 years	1889 (20.49)	64.96 (13.86)	60.25 (13.61)	63.02 (9.62)	5.59 (1.15)
Countries					
Argentina	1266 (13.73)	65.72 (13.36)	60.25 (13.26)	63.47 (9.57)	5.58 (1.01)
Brazil	2000 (21.70)	65.89 (13.67)	60.10 (13.37)	63.51 (9.16)	5.05 (1.20)
Chile	879 (9.54)	62.22 (15.17)	60.27 (12.86)	61.42 (10.33)	5.65 (1.08)
Colombia	1230 (13.34)	65.71 (12.89)	60.26 (13.04)	63.47 (9.04)	5.55 (1.11)
Costa Rica	798 (8.66)	65.67 (13.15)	60.30 (14.15)	63.46 (9.41)	5.82 (1.08)
Ecuador	800 (8.68)	65.62 (12.81)	60.35 (13.89)	63.45 (8.70)	6.42 (0.92)
Peru	1113 (12.07)	65.84 (14.24)	60.14 (14.38)	63.50 (9.23)	5.73 (0.97)
Venezuela	1132 (12.28)	61.92 (11.73)	60.25 (14.36)	61.23 (8.67)	5.62 (1.08)
Socio-economic level					
High	880 (9.55)	67.21 (13.95) +	58.52 (13.70) +	63.91 (9.24)	5.82 (1.54) +
Middle	3542 (38.42)	65.84 (13.87) +	59.77 (13.39) +	63.04 (9.21)	5.63 (1.12) +
Low	4796 (52.03)	63.80 (13.58) +	60.86 (13.74) +	62.66 (9.27) #	5.50 (1.15) +
Weight status					
Underweight	306 (3.32)	64.48 (13.01)	63.10 (12.72)	63.89 (9.13)	5.58 (1.10)
Normal weight	3420 (37.10)	64.70 (13.51)	60.67 (13.58)	63.09 (9.14)	5.58 (1.14)
Overweight	3167 (34.36)	65.36 (13.78)	59.99 (13.65)	63.15 (9.19)	5.61 (1.14)
Obese	2315 (25.11)	64.89 (14.21)	59.47 (13.69)	62.65 (9.35)	5.57 (1.12)
Waist circumference					
Below	6302 (68.37)	65.03 (13.63)	60.48 (13.68)	63.16(9.25)	5.60 (1.14)
Above	2905 (31.51)	64.80 (13.96)	59.65 (13.47)	62.69(7.37)	5.55 (1.13)
Neck circumference					
Below	5889 (63.95)	65.31 (13.65)	60.45 (13.49)	60.70 (9.27)	5.59 (1.14)
Above	3320 (36.05)	64.35 (13.96)	59.80 (13.82)	62.51 (9.33)	5.57 (1.13)

Data are the mean (standard deviation). *** Significant differences between male and female, *p* < 0.001. + All socio-economic levels differed significantly among each other, Bonferroni: *p* < 0.05. # Significantly different from the other two socio-economic levels, Bonferroni: *p* < 0.05.

## Supplementary Materials

The following are available online at https://www.mdpi.com/2072-6643/11/7/1605/s1, Table S1: Dietary patterns in individuals residing in urban areas of Latin American countries, according to socioeconomic level (*n* = 9218). Table S2: Multiple linear regression models.
