# Supplementary material for: Diet Quality and Diet Diversity in Eight Latin American Countries: Results from the Latin American Study of Nutrition and Health (ELANS)
Source: Nutrients. 2019 Jul 15;11(7):1605. doi: 10.3390/nu11071605 (PMC6682987; doi:10.3390/nu11071605)
Supplement: Supplementary file 1 [file nutrients-11-01605-s001.pdf]

nutrients

MDPI

Article

Diet quality and diet diversity in eight Latin American countries: Results from the Latin American Study of Nutrition and Health (ELANS)

Table S1. Dietary patterns in individuals residing in urban areas of Latin American countries, according to socioeconomic level. (n = 9218).

Country	Socio-economic level	n (%)	Score based on greater consumption of healthy dietary items Mean (SD)	p^1	Score based on lower consumption of unhealthy dietary items Mean (SD)	p^1	Score based on healthy and unhealthy dietary items Mean (SD)	p^1
Argentina	High	65 (5.13)	69.11 (14.61)[a]		60.83 (12.80)		65.70 (9.55)	
	Middle	585 (42.21)	56.73 (14.50)[a]	0.003	59.36 (13.20)	0.089	63.70 (9.30)	0.068
	Low	616 (48.66)	64.41 (13.84)[b]		61.03 (13.32)		63.01 (8.61)	
Brazil	High	169 (8.45)	65.70 (14.06)[a]		58.77 (12.55)[a,b]		63.90 (9.94)	
	Middle	915 (45.75)	66.50 (13.38)[a]	0.013	59.42 (13.20)[a]	0.036	63.64 (9.27)	0.658
	Low	916 (45.80)	64.88 (13.81)[b]		61.01 (13.64)[b]		63.29 (8.90)	
Chile	High	80 (9.10)	67.73 (14.54)[a]		59.10 (11.79)		64.18 (9.35)[a]	
	Middle	388 (44.14)	64.31 (59.17)[a]	0.000	60.67 (13.19)	0.630	62.81 (11.18)[a]	0.000
	Low	411 (46.76)	59.17 (15.17)[b]		60.13 (12.89)		59.56 (10.34)[b]	
Colombia	High	67 (5.45)	60.77 (13.61)[a]		69.01 (12.74)		65.61 (9.30)	
	Middle	384 (31.22)	59.66 (12.95)[a,b]	0.028	66.48 (12.87)	0.463	63.67 (9.30)	0.092
	Low	779 (63.33)	60.51 (13.04)[b]		65.05 (12.86)		63.18 (8.87)	
Costa Rica	High	108 (13.53)	67.00 (13.36)		58.70 (13.81)		63.58 (9.66)	
	Middle	428 (53.63)	66.14 (13.17)	0.171	60.27 (14.09)	0.326	63.72 (9.30)	0.662
	Low	262 (32.83)	64.35 (13.00)		61.00 (14.38)		62.97 (9.51)	
Ecuador	High	104 (13.00)	67.23 (13.60)		59.18 (14.04)		63.91 (9.66)	
	Middle	297 (16.78)	65.74 (12.97)	0.337	60.22 (13.79)	0.465	63.72 (9.30)	0.626
	Low	399 (49.88)	65.10 (12.47)		61.00 (14.38)		62.97 (9.51)	

Peru	High	225 (20.22)	67.67 (14.10)		55.09 (14.41) [a]		62.37 (9.62) [a]	
	Middle	355 (31.90)	65.45 (14.16)	0.189	59.50 (13.81) [b]	0.000	63.01 (9.30) [a,b]	0.006
	Low	533 (47.89)	65.41 (14.33)		62.70 (14.44) [c]		64.30 (8.95) [b]	
Venezuela	High	62 (5.48)	61.13 (12.05)		63.27 (14.48)		62.01 (9.37)	
	Middle	190 (16.78)	60.74 (11.76)	0.157	59.77 (13.23)	0.160	60.34 (8.31)	0.153
	Low	880 (77.74)	62.23 (11.72)		60.13 (14.57)		61.37 (8.69)	

Table S2. Multiple linear regression models.

Dependent variable: Total dietary quality score (DQS)						
Predictors	*R*	*R^2 adjusted*	*DF*	*F*	*P value*	*Durbin-Watson*
Healthy DQS	0.797	0.635	1, 9216	16004.86	0.0001	2.023
Unhealthy DQS*	0.505	0.255	1, 9216	3162.47	0.0001	1.859
Dependent variable: Dietary diversity score (DDS)						
Predictors	*R*	*R^2 adjusted*	*DF*	*F*	*P value*	*Durbin-Watson*
Healthy DQS	0.171	0.029	1, 9216	275.98	0.0001	1.835
Unhealthy DQS*	0.120	0.014	1, 9216	1.209	0.227	1.860
Dependent variable: DDS						
Predictor: Healthy DQS	*R*	*R^2 adjusted*	*DF*	*F*	*P value*	*Durbin-Watson*
Men	0.184	0.034	1, 4407	154.91	0.0001	1.820
Women	0.158	0.025	1, 4807	123.69	0.0001	1.858
Dependent variable: DDS						
Predictor: Healthy DQS	*R*	*R^2 adjusted*	*DF*	*F*	*P value*	*Durbin-Watson*
Age interval: 5-19	0.162	0.026	1, 1221	32.82	0.0001	1.877
Age interval: 20-34	0.172	0.029	1, 3477	105.57	0.0001	1.813
Age interval: 35-49	0.169	0.029	1, 2625	77.60	0.0001	1.896
Age interval: 50-65	0.175	0.031	1, 1887	59.50	0.0001	1.932
Dependent variable: DDS						
Predictor: Healthy DQS	*R*	*R^2 adjusted*	*DF*	*F*	*P value*	*Durbin-Watson*
Socio-economic level low	0.155	0.024	1, 4794	118.78	0.0001	1.813
Socio-economic level middle	0.174	0.030	1, 3540	110.63	0.0001	1.886
Socio-economic level high	0.183	0.033	1, 878	30.31	0.0001	1.899

Dependent variable: DDS

Predictor: Healthy DQS	*R*	*R^2 adjusted*	*DF*	*F*	*P value*	*Durbin-Watson*
Ecuador	0.077	0.006	1, 798	4.719	0.03	2.004
Argentina	0.086	0.007	1, 1264	9.37	0.002	1.860
Chile	0.140	0.020	1, 877	17.52	0.0001	1.937
Costa Rica	0.140	0.020	1, 796	15.92	0.0001	1.855
Peru	0.150	0.022	1, 1111	25.51	0.0001	1.860
Colombia	0.171	0.029	1, 1228	37.05	0.0001	1.838
Venezuela	0.200	0.040	1, 1130	47.26	0.0001	2.011
Brazil	0.287	0.082	1, 1998	179.05	0.0001	1.860

Dependent variable: DDS

Predictor: Healthy DQS	*R*	*R^2 adjusted*	*DF*	*F*	*P value*	*Durbin-Watson*
Weight status, normal	0.189	0.036	1, 3724	137.40	0.0001	1.887
Weight status, overweight	0.159	0.025	1, 5480	142.30	0.0001	1.845
Waist circumference, below	0.190	0.036	1, 6300	235.07	0.0001	1.887
Waist circumference, above	0.129	0.017	1, 2903	49.49	0.0001	1.805

R: regression coefficient. R2 adjusted: it refers to the portion of variation in the dependent variable explained by the independent variable corrected by the number of variables added to the model. DF: degree of freedom. F: Fisher's test. P: significance value. Durbin-Watson statistic is a test for autocorrelation in the residuals in regression analysis. Values around 2 indicate that errors are uncorrelated. * The stepwise method excluded these variables from the regression model.
